# [Retracted] Chinese herbal formula QHF inhibits liver cancer cell invasion and migration

**DOI:** 10.3892/etm.2025.12890

**Published:** 2025-05-19

**Authors:** Tao Chen, Quan Wang, Yunxiao Li, Hefei Huang, Wei Hu

Exp Ther Med 11:2413–2419, 2016; DOI: 10.3892/etm.2016.3247

Following the publication of the above article, a concerned reader drew to the Editor’s attention that there were potential anomalies associated with the Transwell migration and invasion assay data shown in Figs. 2, 3 and 7; essentially, patternings of subgroups of cells within the images were very similar, to the extent that it was difficult to conceive of the similarities as being coincidental. Moreover, the scratch wound assay data shown in Fig. 1A and C on p. 2415 were noted to be overlapping, and the western blot data shown in Figs. 4 and 6 for the ERK and JNK experiments respectively on p. 2417 were strikingly similar, suggesting that these data were derived from the same original source.

After having conducted an internal investigation of the data in this paper, the Editor of *Experimental and Therapeutic Medicine* has judged that the potentially anomalous presentation of the strikingly similar groupings of cells in Figs. 2, 3 and 7 was too extensive that these features could have been easily attributed to coincidence, in addition to the incorrect assembly of Figs. 1, 4 and 6. Therefore, the Editor has decided that this article should be retracted from the publication on the grounds of an overall lack of confidence in the data. The authors were asked for an explanation to account for these concerns, but the Editorial Office did not receive a reply. The Editor sincerely apologizes to the readership for any incovenience caused, and we thank the reader for bringing this matter to our attention.

